# Traumatic Spinal Injury among Patients with Spinal Injuries Admitted to the Spine Unit of a Tertiary Care Centre: A Descriptive Cross-sectional Study

**DOI:** 10.31729/jnma.6850

**Published:** 2022-04-30

**Authors:** Krishna Prasad Paudel, Sunil Panta, Shrawan Kumar Thapa, Sushil Thapa

**Affiliations:** 1 Department of Orthopaedics, Bharatpur Hospital, Bharatpur, Chitwan, Nepal

**Keywords:** *falls*, *polytrauma*, *spinal cord injuries*, *trauma*

## Abstract

**Introduction:**

Traumatic spinal injury is a major source of morbidity and mortality throughout the world. The number of spinal injuries is growing annually but epidemiological and demographic features may be different in different regions. This study aims to find out the prevalence of traumatic spinal injury among patients with spinal injuries admitted to the spine unit of a tertiary care centre.

**Methods:**

This was a descriptive cross-sectional study was done on a total of 102 traumatic spinal injury patients admitted to the spine unit of a tertiary care centre from 1^st^ June, 2019 to 31^st^ May, 2021 after receiving ethical approval from the Institutional Review Committee (Reference number: 077/78-09). Demographic details, mode of injury, morphology, patterns of fractures, neurological level, and management methods in the hospital were recorded. Convenience sampling was done. Data were analysed using the Statistical Package for the Social Science version 24.0. Point estimate at 95% Confidence Interval was calculated along with frequency and percentages for binary data.

**Results:**

Among 130 spinal injury patients, the prevalence of traumatic spinal injury was found to be 102 (78.46%) (71.39-85.53 at 95% Confidence Interval). The most common mode of spinal injury was due to falls in 80 (78.43%) cases.

**Conclusions:**

The prevalence of traumatic spinal injury was higher when compared to the other studies done in similar settings.

## INTRODUCTION

The incidence and prevalence of traumatic spinal injury are increasing with an increase in fall injury, roadside accidents, etc. The majority of the data about spinal injuries are from the western world or countries with advanced trauma care.^1^ In a systematic review, the overall global incidence of Traumatic Spinal Injury (TSI) was 10.5 cases per 100,000 persons.^2^ Traumatic spinal cord injury is a subset of traumatic spinal injury.^2^ The annual incidence of Spinal Cord Injury (SCI) is approximately 54 cases per million people in the United States, excluding those who die at the location of the incidence.^3^

Data from developing countries like Nepal are scarce due to the lack of a trauma database. Understanding the epidemiology of spinal injury helps to prevent trauma itself, identify risk factors, reduce disability, prevent mortality, and help to develop policy and treatment strategies.

This study aims to find out the prevalence of traumatic spinal injury among patients with spinal injuries admitted to the spine unit of a tertiary care centre.

## METHODS

A descriptive cross-sectional study was done at the Department of Orthopaedics, Bharatpur Hospital from 1^st^ June, 2019 to 30^th^ May, 2021. The ethical approval was taken from the Institutional Review Committee (Reference number: 077/78-09) of the hospital. All the patients with spinal injuries admitted to the spinal ward were included in the study. Those patients with incomplete data and patients with head injuries were excluded from this study. A convenience sampling was done.

The sample size was calculated using the following formula:

n = (Z^2^ × p × q) / e^2^

  = (1.96^2^ × 0.5 × 0.5) / 0.09^2^

  = 119

Where,

n = minimum required sample size
Z = 1.96 at 95% Confidence Interval (CI)
p = prevalence of traumatic spinal injury was taken as 50% for maximum sample size
q = 1-p
e = margin of error, 9%

The minimum required sample size was 119. However, a total of 130 sample size was taken for the study. The proforma was filled with the demographic profile of the patient, mode of injury, neurology according to the American Spinal Injury Association Score (ASIA), vertebral level and type of injury, management methods, surgical techniques, rehabilitation, inhospital complications, and recovery.

The diagnosis of spinal injury was made with history, clinical examination, X-ray spine, Computed Tomography (CT) spine and Magnetic Resonance Imaging (MRI) spine. Data were collected from the filled proformas, medical records of the patients admitted to the spinal ward and operation records. The data was collected and descriptive analysis was done through the Statistical Package for the Social Science version 24.0. Point estimate at 95% Confidence Interval was calculated along with frequency and percentages for binary data.

## RESULTS

**Figure 1:**
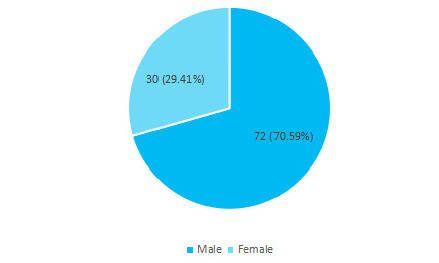
Sex-wise distribution in percentage (n= 102).

Among 130 spinal injury patients, the prevalence of traumatic spinal injury was found to be 102 (78.46%) (71.39-85.53 at 95% Confidence Interval). This study showed 72 (70.59%) were males and 30 (29.41%) were females. The male-female ratio was 2.4:1 (Figure 1).

**Figure 2:**
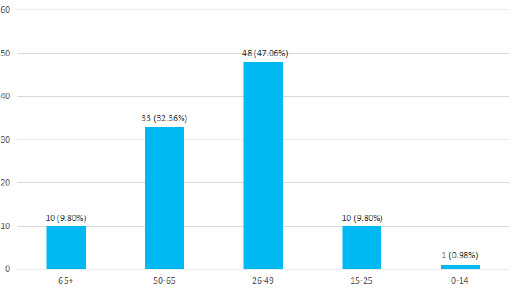
Age-wise distribution of patients (n= 102).

The ethnic distribution of these patients showed 50 (49.02%) were Janajati, 29 (28.43%) were Brahmin, and Chhetri, 20 (19.61%) were Dalit, 2 (1.96%) were Madhesi, and 1 (0.98%) belonged to the Muslim community. The mean age was 44.06±15.23 years. The commonest occurrence of the injury, 81 (79.41%) was in the working group of people i.e. from 26 to 65 years of age (Figure 2).

The most common mode of spinal injury was due to falls in 80 (78.43%) cases. The modes of injuries showed 45 (44.12%) were fall from heights, 32 (31.37%) fell from a tree, 13 (12.75%) were roadside accidents, 3 (2.94) were wildlife injuries, 3 (2.94%) were fall from a cliff, 2 (1.96%) had a history of domestic violence and got a complete cervical cord injury, 2 (1.96%) were committed to suicide and 2 (1.96%) were others mode of injuries (Table 1).

**Table 1:** Mode of spinal injuries (n= 102).

Mode of injury	n (%)
Fall from height	45 (44.12)
Fall from tree	32 (31.37)
Roadside accident	13 (12.75)
Fall from cliff	3 (2.94)
Wildlife injury	3 (2.94)
Violence	2 (1.96)
Suicidal	2 (1.96)
Others	2 (1.96)

The cervical region 45 (44.12%) was the most common spinal region involved and followed by the lumbar region (Table 2).

**Table 2:** Spinal region involved (n = 102)

Spinal Region	n (%)
Cervical	45 (44.12)
Dorsal	24 (23.53)
Dorsolumbar	8 (7.84)
Lumbar	25 (24.51)

**Figure 3:**
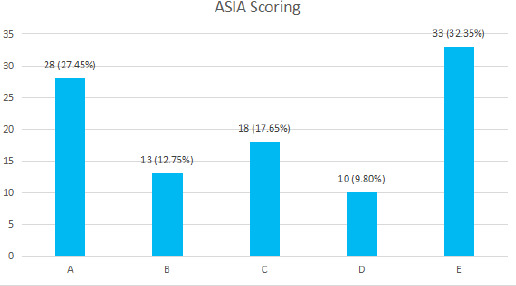
Neurological grading of patients (n= 102).

ASIA scoring was done after assurance of recovery from spinal shock. The persons with normal neurology were considered only to have a traumatic spinal injury and no cord injury as Magnetic Resonance Imaging was not done in all cases. Ten patients had multiple fractures and two patients had associated other organ injuries. Mortality was seen in 5 (4.9%) admitted patients. Two patients with complete cervical cord injury died in the intensive care unit in the preoperative period and two others with cervical cord injuries died in the post-operative period (Figure 3).

Fifty-six (54.9%) of patients were operated and 41 (40.2%) were managed conservatively. Three (2.9%) either died or were referred. The spinal shock was found over in all cases before surgery. Among 41 patients managed conservatively, 23 (56.09%) had dysfunction of the bowel and bladder. Fifty-one (50%) among 102 injured patients had bowel bladder dysfunction.

By the frequency, 13 (12.74%) patients had an L1 vertebral fracture, and 11 (10.70%) patients had vertebral fracture levels at C4, C5, and C5, C6 each. Ten (9.8%) patients with fracture levels of C6 and C7. Other common levels of injury were L2 vertebra fractures in 9 (8.8%) patients and D12-L1 fractures in 5 (4.9%) patients. The commonest type of vertebral fracture observed in these patients was burst fracture in 33 (32.35%), followed by fracture-subluxation was 25 (24.50%), compression fracture was 21 (20.58%), fracture-dislocation in the cervical and dorso-lumbar spine was 11 (10.78%). Only cord contusion without vertebral fracture was seen in 10 (9.8%) including 4 (3.9%) with central cord syndrome. In 2 (1.9%) patients, there was only traumatic disc rupture without vertebral fracture causing a neurological compromise in the cervical spine.

Eighteen (40%) cases of 45 cervical spine injuries were operated by anterior approach. Sixteen (88.89%) were anterior cervical discectomy and fusion (ACDF) and two (11.11%) were anterior cervical corpectomy and fusion (ACCF). Three (6.67%) cases were stabilised and fused by posterior approach i.e. 2 (66.67%) by lateral mass screws and 1 (33.33%) by interspinous wiring. All 57 (55.88%) dorsal, dorso-lumbar, and lumbar vertebrae fractures were stabilised posteriorly by a pedicle screw system. Out of 97 patients managed in the hospital, only 32 (32.98%) patients turned for follow-up at least one time after discharge. Only 10 (9.8%) patients with SCI had gone for scheduled 6 weeks of spinal rehabilitation. Six (5.8%) patients with complete SCI had repeated follow-ups with sacral and gluteal pressure sores.

## DISCUSSION

Management of traumatic spinal injury (TSI) in developing countries differs from countries with advanced trauma care.^1^ The emergency trauma team is well equipped in extricating victims from the site of the accident. The medical team reaches the accident site within an hour either by road or air ambulances which are staffed with basic life support trained personnel and equipment. In contrast, the scenario is painful in Nepal where injudicious methods of the handling of patients in injury sites and their transport, as learned from the study proformas, often make incomplete spinal cord injury into a complete one. In-hospital handling of suspected spinal injury cases in emergency units of the majority of hospitals is also suboptimal. It is because of the unawareness of the public, ambulance drivers, and health care workers in the country about spinal cord injury and its consequences.

From an average of 26.8 years in Turkey to 55.5 years in the USA, the mean age of spinal injury differs in many countries.^4^ In a hospital-based study in India, the largest number of patients was in the age range of 2039 years closely followed by that of 40-59 years.5 In our study, 48 patients were in the age group of 26 to 49 years and 33 patients in the 50-65 years of age group. The mean age was 44.06 years and this reflects that spinal injury occurs in people in the productive age group who have a major contribution to their family and society.

In the US 80.7% of SCI occurred in males, and a study from Bangladesh revealed 84% to be male patients.^1^ In our study, the incidence of spine trauma was also more in males. Unlike the previous studies from the US and Bangladesh, the incidence of TSI in male patients was 70.59%. The 29.41% incidence in females may have been due to their compulsion in hilly and rural areas of Nepal to climb trees to chop wood for firewood and fodders of cattle.

Unlike the western world where road traffic accidents are the major cause of spine injury, road traffic accidents constituted only 12.8% of spine trauma in this study. As victims from motor vehicle accidents sustaining polytrauma used to go to nearby medical colleges where emergency trauma service is better than this government hospital, the proportion of roadside accidents was found less in this study. In a study from the national trauma centre in Nepal, fall injury was the most frequent mode of injury representing 83.3% followed by MVA.^6^ With the increasing number of road traffic accidents, Motor Vehicle Accident (MVA) has been projected as a major aetiology of spinal cord injury in Nepal and other developing countries.^7^ MVA contributes to 59.5% of spinal cord injuries in other Asian countries.^8^

Fall injuries are the leading cause of spine trauma in developing countries.^9^ In this hospital, 76.4% of patients fell from height including falls from trees sustaining vertebral column injuries. Fall from height other than the tree was from a staircase, building, bridge, etc. It is similar to the studies where the fall was either from a tree or a cliff.^10^ In rural Nepal, wood is still used in the kitchen; therefore, people climb trees to chop the branches. Also, in the countryside, people graze their cattle in the hills and sustain fall injuries. These are preventable causes and programs focusing on preventive strategies should be disseminated in rural areas. Cervical spine injuries (45 out of 102) had higher incidences in this study which usually have grievous consequences leading to quadriparesis or quadriplegia. It is higher than other studies like a hospital-based study where is 36.2% of total patients sustained cervical injuries.^5^

Janjati 50 (49%) and Dalit 20 (19.6%) communities comprised 68.6% of the patients visiting Bharatpur hospital. The proportion of Brahmin-Chhetri in the Nepalese community is higher but their presence among spinal injured people in the hospital was only 29 (28.43%). It could explain that either people from low socioeconomic status came to government hospitals or the injury occurred more in that group of people. Out of Janjati, thirteen patients were Chepang who are indigenous and marginalised people residing in hilly areas of Chitwan and Makawanpur. As Chitwan national park is near Bharatpur hospital, three persons with wildlife injuries presented with TSI.

Management of these patients was comparable to a systematic review and meta-analysis in the United States where 48.8% of persons suffering traumatic spinal injury underwent surgery.^2^ Time taken for surgery from admission was also long i.e. mean time duration of 4.47 days. It is not only because of the late arrival of the injured patients to the hospital but also because of the availability of an operation room for orthopaedics only 3 days a week. It may affect the neurological recovery of the patients.^1^ The Government of Nepal, in 2014, established an insurance scheme in which a spinal cord injured patient is covered with treatment expenses of a maximum of 1 lakh Nepalese rupee.^9^ Expenses more than this have to be paid by the patient. This amount is sufficient to cover investigations, beds, medicines, and surgery but not sufficient for spine implants. This initiative is wonderful for poor Nepalese but still arranging implants is a major cause of delay in the surgery.

The limitation of the study is that it was a retrospective single hospital-based analysis. The patients who were discharged or left the hospital from the emergency are missed from this analysis. Arrival time at the hospital from the incident couldn't be traced out. Similarly, primary treatment at the site of injury and methods of transfer couldn't be traced in the majority of the cases so not mentioned here. There was a limited follow-up of patients so treatment outcome and recovery could not be described.

## CONCLUSIONS

The prevalence of traumatic spinal injury was higher when compared to the other studies done in similar settings. Fall from height was the most common mechanism of injury especially that from a tree and roadside accidents are also increasing in number. Public awareness about the spinal injury; education on primary treatment at the injury site and safe transfer to the hospital are important preventive measures. People should be instructed to use safety harnesses for those whose occupations involve climbing trees/ heights. Road safety measures must be strengthened. A policy and treatment protocol should be prepared by the Ministry of Health with the help of stakeholders to provide ideal and optimal care (both acute and rehabilitation) to traumatic spinal injury patients as well as its prevention.
